# Association of mRNA Levels of IL6, MMP-8, GSS in Saliva and Pyelonephritis in Children

**DOI:** 10.3390/molecules25010085

**Published:** 2019-12-25

**Authors:** Sirma Angelova, Ayshe Salim, Yoana Kiselova-Kaneva, Diana Ivanova, Stefan Peev

**Affiliations:** 1 Department of Pediatric Dentistry, Faculty of Dental Medicine, Medical University of Varna, 9000 Varna, Bulgaria; dsirma_angelova@abv.bg; 2 Department of Biochemistry, Molecular Medicine and Nutrigenomics, Faculty of Pharmacy, Medical University of Varna, 9000 Varna, Bulgaria; ykisselova@abv.bg (Y.K.-K.); divanova@mu-varna.bg (D.I.); 3 Department of Periodontology and Dental Implantology, Faculty of Dental Medicine, Medical University of Varna, 9000 Varna, Bulgaria; stefan.peev@mu-varna.bg

**Keywords:** salivary biomarkers of IL-6, MMP-8, GSS, pyelonephritis, children, mRNA levels, correlation

## Abstract

Nowadays, saliva is a subject of growing scientific interest because of its definite advantages as diagnostic medium. The aim of our study was to investigate the diagnostic potential and reliability of messenger RNAs (mRNAs) of selected genes—interleukin-6 (IL-6), matrix metalloproteinase-8 (MMP-8) and glutathione synthetase (GSS)—as salivary markers in children with diagnosed pyelonephritis and to correlate their levels with typical urine para-clinical indicators of the disease. Analysis of the mRNA levels for IL-6, MMP-8 and GSS in 28 children hospitalized with the diagnosis of pyelonephritis was conducted applying the method of quantitative reverse transcription polymerase chain reaction (RT-qPCR). In the study group (*n* = 28), IL-6 mRNA levels demonstrated 64-fold increase (*p* < 0.001). MMP-8 and GSS mRNA levels were increased in 12 samples in patients with pyelonephritis 3.27 (*p* < 0.01) and 1.94 (*p* < 0.001) times, respectively. We found a strong and significant correlation (*p* < 0.001) between the investigated mRNA for IL-6 and MMP-8, IL-6 and GSS, MMP-8 and GSS. Moderate degree of correlation was established between IL-6 and the typical para-clinical indicator of leucocytes (0.43, *p* < 0.05) and between GSS and leucocytes (0.54, *p* < 0.01). Salivary IL-6, MMP-8 and GSS mRNA levels in combination with urine test analysis could be useful diagnostic tool for the very distributed disorder of pyelonephritis in childhood.

## 1. Introduction

Nowadays there is a constant increase of the scientific interest in the application of minimally invasive diagnostic procedures [1]. Saliva is a biological substrate which includes more than 3000 species of mRNA (messenger RNA), which could serve as biomarkers in regard to various health disorders [2]. Compared to both of the most widely distributed laboratory approaches of blood and urine assays, saliva is characterized by definite advantages. Collection of saliva is a feasible procedure with minimized risk of pathogenic microorganism distribution. In terms of qualitative and quantitative characteristics saliva as a unique combination of organic and non-organic compounds is simplified and slightly fluctuating compared to blood serum [3,4]. Based on the anatomic and physiological specifics of salivary glands, there are real conditions for molecular exchange between blood and acini. Biomarkers of the bloodstream can penetrate through the acinar structures and be excreted into the saliva [5]. 

Urinary tract infections (UTIs) are characterized by high prevalence in different periods of childhood, especially during the stages of early childhood and preschool age [6]. These disorders of the excretory system are ranked second in incidence and clinical manifestation after inflammatory respiratory tract diseases [7,8,9,10]. Nowadays, the concept of personalized medicine and an individually oriented therapeutic approach has a considerable impact upon the issue of susceptibility to UTIs. The individual inflammatory response correlates with the extent of progression of UTIs, clinically manifesting with renal scarring and progressive kidney injury [11]. Many scientific investigations are devoted to clarification of the significance of individual genetic medium of inflammatory reactions. The minimization of tissue morphology and function deterioration is obtainable by determining the adequate indicators underlying acute pyelonephritis and its proper therapy [7,12,13,14]. The diagnosis, monitoring and effective treatment of pyelonephritis provide significant challenges for clinical practice due to its recurrence, increase of antibiotic resistance and the future complications related to the risk of a functional impairment and progression to chronic renal failure [7,12,15,16,17]. A diagnosis of acute pyelonephritis is initially done on the presence of clinical symptoms and para-clinical parameters, including urine analyses. In the context of individual reactivity towards pathological processes along the excretory system, the application of the dipstick urine analysis alone can be insufficient [18]. Dipstick urinalysis is characterized as a conventional diagnostic method, but its implementation is associated to false-positive and false-negative results [19]. It is related to the explicit necessity of new, alternative diagnostic methods and mediums of investigation. On the other hand, the specifics of child’s behavior, respectively very frequent lack of inclination and readiness for collaboration during prognostic, diagnostic and treatment procedures, provoke researchers to seek for the potentials and advantages of saliva as a diagnostic environment. According to profound scientific investigations, saliva-based methods have demonstrated exponential growth in recent decades. Taking into consideration specifics of age, traits of physiological development and growth, dietary regimen in different periods of childhood, saliva ensures for pediatric specialists’ new opportunity for prognosis and diagnosis of systemic disorders [20,21,22,23,24,25]. Implementation of saliva as a diagnostic tool is characterized with no discomfort, no pain, no traumatic injuries to blood vessels and no interventions and is a minimally invasive approach to the patient child. Not to neglect the fact that the collection, storage and application of saliva as a diagnostic substrate is characterized with considerably lower risk for transmissive infections compared to blood tests. The establishment of markers based on saliva can be utilized for evaluation of the extent and course of the inflammatory process. 

Different molecules are involved in the inflammatory process and some of them could serve as reliable biomarkers for the state and progression of specific inflammatory diseases. It is known that the pro-inflammatory cytokine IL-6 is implicated in renal inflammatory diseases. Specific cells of kidney tissue, respectively tubular epithelial cells, mesangial cells, endothelial cells and podocytes, have the potential to secrete IL-6 [26]. Matrix metalloproteinases (MMPs) play an important role in the recruitment of inflammatory cells at the place of inflammation, thus, regulating the inflammatory response [27,28,29]. Matrix metalloproteinase-8 (MMP-8) is a protease mainly expressed by neutrophils [30]. Acute pyelonephritis in rat model is accompanied by increase in oxidative stress markers and tissue damage [31]. In children, total and enzyme antioxidant capacity in combination with clinical parameters could be used as markers for inflammatory and immunological active parenchymal kidney disorders, including pyelonephritis [32]. Most of the studies exploring the diagnostic potential of different salivary biomarkers in renal diseases are focused mainly on chronic renal failure [33,34,35]. On the other hand, the majority of these articles targeted adult population [36,37,38,39,40,41]. The application of non-invasive tools for disease diagnosis and monitoring in children could significantly improve +health care.

## 2. Results

### 2.1. Salivary mRNA Levels of IL-6, MMP-8 and GSS

The investigated salivary biomarkers—IL-6, MMP-8 and GSS—re directly involved in inflammatory process. Scientific literature confirms that they increase in conditions of pyelonephritis—an inflammatory process of the excretory system [42,43,44,45,46,47,48]. The investigation of the combination of these parameters gives more detailed information on the course and extent of the inflammatory process, and also on the individual differences in the progression of the disease.

**Figure 1:**
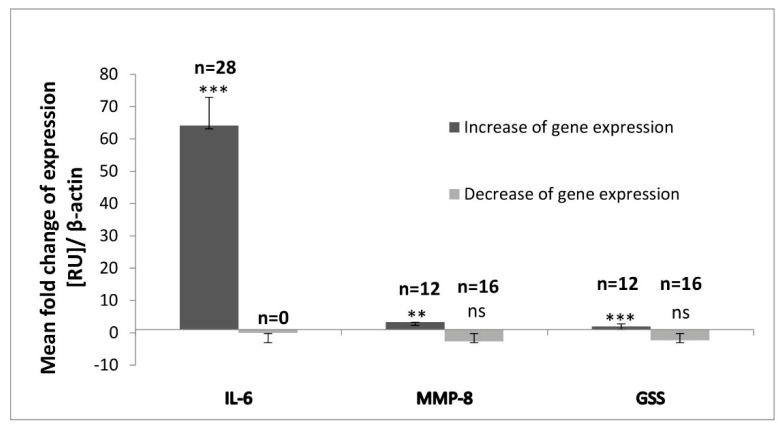
Mean fold-change of interleukin-6 (IL-6), matrix metalloproteinase-8 (MMP-8), glutathione synthetase messenger RNA (GSS mRNA) levels for the total investigated group of 28 children with diagnosed pyelonephritis. All fold changes are presented as mean for the total group versus a control group of nine children with no common health disorders. The expression of each gene of interest in the control group is considered to be equal to 1. Expression levels are presented in relative units (RU) ± SEM and human β-actin was used as endogenous control to normalize mRNA levels of IL-6, MMP-8 and GSS in each sample. ** *p* < 0.01 versus control group; *** *p* < 0.001 versus control group; ns—not significant.

RT-qPCR reaction was performed to quantitatively detect the mRNA levels of IL-6, MMP-8 and GSS in saliva samples. The subject of our study were 28 children suffering with pyelonephritis. A control group of nine children with no common health disorders was also included. We recorded that the mRNA levels of the pro-inflammatory cytokine IL-6 were considerably higher compared to these of MMP-8 and GSS for all 28 children with pyelonephritis. In all samples, IL-6 mRNA levels were elevated as compared to the healthy group. The increase of IL-6 expression for the total group was 64-fold compared to the control group (*p* < 0.001) (Figure 1).

The investigated group of 28 children with pyelonephritis could be split into two subgroups, based on the results obtained for the levels of the three investigated salivary parameters. For MMP-8, we have established a statistically significant 3.27-fold increase of the gene expression only among 42.86% (*n* = 12) of all the investigated children (*p* < 0.01) (Figure 1). The other 55.14% (*n* = 16) showed a statistically non-significant 2.66-fold decrease of the expression (Figure 1). Pearson’s correlation coefficient between the indicators MMP-8 and IL-6 equaled to 0.75 (*p* < 0.001). This indicates a strong correlation between both indices.

**Figure 2:**
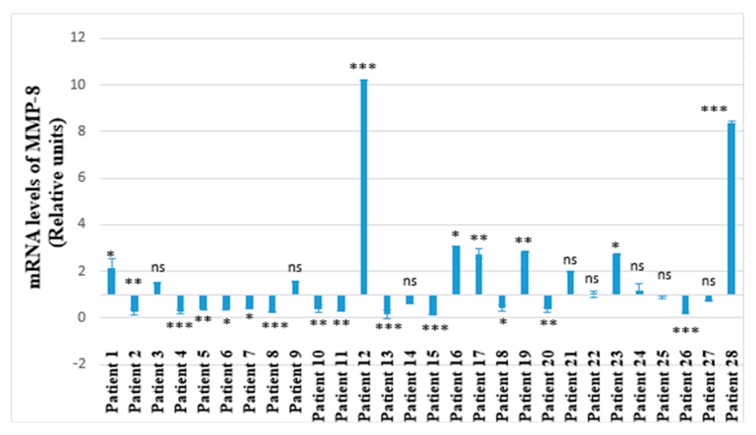
MMP-8 mRNA levels in saliva of 28 children with diagnosed pyelonephritis. Expression levels are presented in relative units ± SEM versus human β-actin as endogenous control. * *p* < 0.05 versus control group; ** *p* < 0.01 versus control group; *** *p* < 0.001 versus control group; ns—not significant.

**Figure 3:**
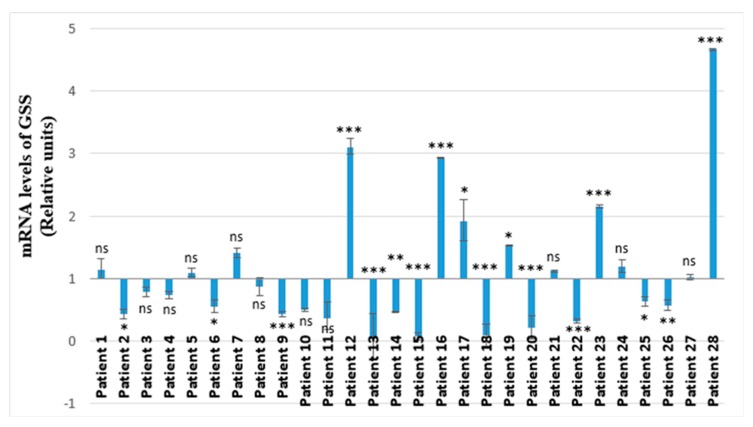
GSS mRNA levels in saliva of 28 children with diagnosed pyelonephritis. Expression levels are presented in relative units ± SEM versus human β-actin as endogenous control. * *p* < 0.05 versus control group; ** *p* < 0.01 versus control group; *** *p* < 0.001 versus control group; ns—not significant.

**Figure 4:**
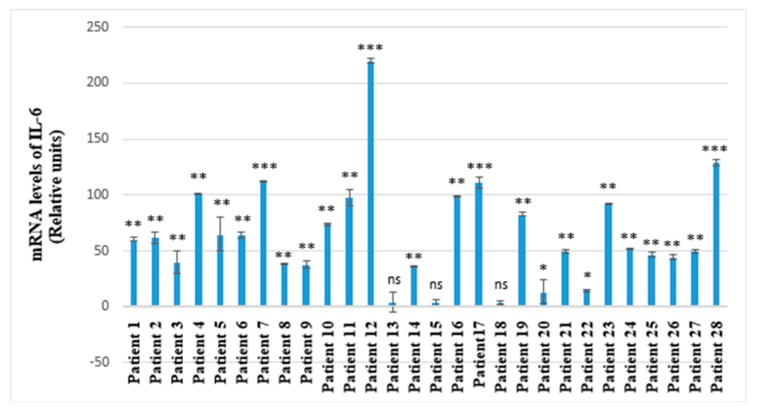
IL-6 mRNA levels in saliva of 28 children with diagnosed pyelonephritis. Expression levels are presented in relative units ± SEM versus human β-actin as endogenous control. * *p* < 0.05 versus control group; ** *p* < 0.01 versus control group; *** *p* < 0.001 versus control group; ns—not significant.

A 1.94-fold increase of GSS expression with statistical significance was established for 41.38% (*n* = 12) of all of the investigated children (*p* < 0.001) (Figure 1). The other 58.62% (*n* = 16) marked by reduction of the GSS expression were without any statistical significance (*p* = 0.062) (Figure 1). Almost all of the patients with increase of the MMP-8 expression (Figure 2) also showed elevation of the GSS expression (Figure 3) and vice versa. The same patients showed also higher IL-6 expression levels compared to the others (Figure 4). Variations were established only in the results without statistical significance. We found a strong Pearson’s correlation (0.75, *p* < 0.001) between GSS and IL-6, and also a strong correlation between GSS and MMP-8 equaling to 0.86 (*p* < 0.001). 

Among all 28 participants with diagnosed pyelonephritis, nine showed considerably high mRNA levels of IL-6, MMP-8 and GSS (Patients № 1, 12, 16, 17, 19, 21, 23, 24, 28). Six of these nine patients showed statistically significant results for the three investigated parameters (patients 12, 16, 17, 19, 23, 28) (Figure 2, Figure 3 and Figure 4). Two of the patients (patient 12 and patient 28) had considerably higher mRNA levels for all of the investigated genes (IL-6, MMP-8, GSS) (Figure 2, Figure 3 and Figure 4). 

### 2.2. Comparison between the Investigated Salivary Biomarkers and Urine Analysis Indicators

Concerning both patient 12 and 28, we obtained not only the highest mRNA levels of all the investigated salivary biomarkers, but we also established the greatest values of para-clinical indicators—leucocytes and blood in urine analyses—compared to all the participants (Figure 2, Figure 3 and Figure 4, Table 1). Patient 12 showed the highest mRNA levels of IL-6 and MMP-8 and a very high level of GSS (Figure 2, Figure 3 and Figure 4). This patient was indicated as a primary patient, with initial onset of pyelonephritis at the time of the investigation. Regarding the para-clinical indicators—blood and leucocytes in urine assay—patient 12 also had the highest values of both of these parameters (250 Ery/µL; 500 Leuc/µL) (Table 1). For patient 28, we have established IL-6 mRNA levels equal to 128,898 relative units, for MMP-8: 8383 relative units and for GSS: 4671 relative units (Figure 2, Figure 3 and Figure 4). In addition, participant 28 had the highest level of leucocytes in urine assay (500 Leuc/µL) and 50 Ery/µL hemolysis for blood (Table 1). 

### 2.3. Correlation between Salivary Biomarkers and Para-Clinical Indicators from Urine Assay

To evaluate the diagnostic potentials of these salivary markers, we calculated Pearson’s correlation coefficient for IL-6, MMP-8 and GSS and the para-clinical indicators—leucocytes, blood and proteins from urine assays—among the group of 28 children. A moderate degree of correlation was established between IL-6 and leucocytes (0.43, *p* < 0.05) and between GSS and leucocytes (0.54, *p* < 0.01) (Table 2). These results are definitely indicative for the prognostic and diagnostic significance of both of these salivary markers in children suffering from pyelonephritis. Slight degree of correlation without statistical significance was assessed between the parameters IL-6 and blood (0.15, *p* = 0.4459) and between GSS and blood (0.20, *p* = 0.2998). Simultaneously, a similar slight degree of correlation without statistical significance was registered between the salivary MMP-8 and proteins in urine samples, amounting to 0.16 (*p* = 0.4236) (Table 2).

## 3. Discussion

Saliva has a lot of advantages as a biological fluid in the laboratory diagnostics, especially among children. It is easy to collect and rich in different analytes, including protein, DNA and RNA originating from different body parts [49]. Care should be taken in its collection and subsequent storage to limit pre-analytical variations and to maintain bioorganic molecules integrity [50]. The para-clinical indicators commonly used to diagnose pyelonephritis in biological fluids such as urine and blood should be collated to the investigated salivary markers to estimate their diagnostic and prognostic potential and the reliability of saliva as a diagnostic medium. 

In the present study, we have examined the mRNA levels of IL-6, MMP-8 and GSS using saliva as an emerging, non-invasive and perspective medium for precise diagnosis of acute pyelonephritis in children. Based on the investigation of the mRNA levels of these three parameters, we recorded that the level of IL-6 was considerably higher compared to MMP-8 and GSS (Figure 1). This result is related to the fact that the indicator IL-6 is one of the most significant pro-inflammatory factors inducing acute inflammatory response of the organism in state of pyelonephritis. Scientific literature confirms that children affected by acute pyelonephritis are characterized with high serum or urine levels of IL-6, which can serve as an indicator of renal damage and is a parameter related to its progression [42,43,44,45,46,47,48].

On the other hand, it was expected that the highest levels of IL-6, MMP-8 and GSS were established among two of the patients, 12 and 28 (Figure 2, Figure 3 and Figure 4). According to personal data based on anamnesis vitae, patient 12 was suffering from recurrent infectious diseases. The latter led to frequent intake of wide spectrum antibiotics and non-steroid anti-inflammatory drugs. The prolonged application of these medicines exercises a strong impact upon the immunological reactivity of the organism. The persisting modifications of immune system functionality are associated with high levels of salivary IL-6 and related to a high level of salivary MMP-8. Our results based on saliva as a diagnostic environment showed diagnostic and prognostic potentials comparable to the capacity of the same markers in other biological media in various common health disorders, including such affecting the excretory system [40,42,45,51]. The obtained highest levels when investigating leucocytes and blood in the urine assay of patient 12 corresponded to the highest levels of salivary biomarkers IL-6, MMP-8 and GSS in the state of disturbed immunological equilibrium (Table 1, Figure 2, Figure 3 and Figure 4). This ascertains the diagnostic and prognostic value of these salivary factors. The other participant in the study with established high levels of salivary factors IL-6, MMP-8 GSS, identified as patient 28, had a congenital anomaly—double right kidney and right urethra (Table 1, Figure 2, Figure 3 and Figure 4). This child was genetically predisposed and had been suffering from epilepsy for a period of almost 10 years. The medicine-controlled epilepsy in combination with congenital renal anomaly was related to explicit manifestation of the inflammatory process in pyelonephritis marked with high levels of the para-clinical indicators blood and leucocytes in urine. These interrelations confirm the already established diagnostic and prognostic significance of the investigated salivary biomarkers in pyelonephritis [52,53,54]. 

We established a strong correlation between the salivary indicators IL-6 and MMP-8. These parameters are associated with the already confirmed functions of MMP-8 in conditions of acute inflammation [30], including in kidney tissue [47]. Matrix metalloproteinases (MMPs) play an important role in the recruitment of inflammatory cells at the place of inflammation, thus, regulating the inflammatory response [27,28,29]. These endopeptidases are associated not only to the normal physiological activity, but are also related to the pathogenesis of kidney diseases [47,55]. The salivary biomarker MMP-8 has a considerable impact not only upon the intensity of inflammation of oral cavity structures, but also upon the dynamics of inflammatory reactions on systemic level [56,57,58]. The obtained results for MMP-8 are associated with the formation of two subgroups among the examined children suffering from pyelonephritis, based on their mRNA levels. Namely, there is a tendency of reduction or enhancement of the gene expression of MMP-8, as compared to the control group of nine healthy children (Figure 2). The obtained results should be interpreted in the conditions of the common health state. Recurrent inflammatory diseases, genetic predisposition and clinical manifestation of common health disorders exercise strong impact upon the immunological response on an individual level. The latter is related to the severity of inflammatory reactions and illustrated on one hand by the levels of investigated salivary parameters, and determined by typical para-clinical indicators in urine on the other [8]. This study showed that MMP-8 has an impact on the pathogenesis and recovery from ischemic acute kidney injury, by potential neutrophil recruitment to the site of injury [47]. 

We established a direct relationship between the elevated mRNA levels of IL-6, MMP-8 and GSS among six of the patients, namely patients 12, 16, 17, 19, 23, 28 (Figure 2, Figure 3 and Figure 4). This interrelation is based on the fact that MMP-8 is a protease mainly expressed by neutrophils and is implicated in inflammation as regulate neutrophil survival [59]. In 83.33% of the representatives of this subgroup, similar abdominal echography characteristics have been established. Namely, in five of these six children, a bilateral drainage disorder on the level of pyelon was diagnosed (Table 1). Another important finding in our study is that the same participants showed considerable increase of the levels of all of the investigated markers—IL-6, MMP-8 and GSS (Figure 2, Figure 3 and Figure 4). This confirms the strong interrelation between these indicators and determines their reliability as diagnostic markers for pyelonephritis, especially when exploring their simultaneous manifestation in a pathological condition. 

The established moderate degree of correlation between IL-6 and leucocytes, moderate degree of correlation between GSS and leucocytes, slight degree of correlation between IL-6 and blood and GSS and blood, as well as between MMP-8 and proteins are principally associated with the pathological alteration and pathophysiological traits of pyelonephritis. Compared to chronic renal failure, the excretory system disorder pyelonephritis is not accompanied by the major impact of MMP-8 upon epithelial architectonics and function. The low rate of impingement of the epithelial tissue in the state of pyelonephritis corresponds to the obtained results illustrating a correlation between blood in urine on one hand, and IL-6 and GSS in saliva, on the other [60]. In the context of pathological morphology and pathophysiological characteristics, the condition of acute inflammation is characterized by an initial phase of alteration, with deterioration of capillary permeability, respectively. The impaired function of capillaries correlates with the subsequent state of exudation. The specific characteristics of an acute inflammatory process correspond to the blood and leucocytes levels in urine assays in patients with diagnosed pyelonephritis. In comparison with the state of chronic renal failure, pyelonephritis does not explicitly manifest with severe disturbance of glomerular filtration, respectively, no high values of proteins in urine samples of the investigated patients have been established [61].

Both the established significant correlation between GSS and IL-6 and the strong correlation between GSS and MMP-8 are related to the fact that oxidative stress is also influenced in inflammatory processes [62,63,64]. Reactive oxygen species (ROS) are released and accumulated at the site of inflammation. Metabolites of arachidonic acid, cytokines and chemokines act by further recruiting inflammatory cells to the site of damage and producing more reactive oxygen species [65]. It is known that GSS is ubiquitously expressed in kidney, colon and other tissues [64]. GSS (GS, EC 6.3.2.3, also known as GSH synthase) catalyze the second step of glutathione biosynthesis [63]. A study found that increases in pro-inflammatory cytokines resulted in an increase of free radicals, targeted by free GSH in host cells [62]. In our study we found a correlation between the highest mRNA levels for IL-6 observed in patients 12, 16, 17, 19, 23, 28 and also the highest levels for GSS established for the same participants. In vitro analyses demonstrate increased expression of the first enzyme from glutathione biosynthesis—GCL in cases of local inflammation [48]. GCL and GSS are regulated coordinately by oxidative stress [66]. The above findings explain the highest mRNA levels of GSS observed in patients with the highest mRNA levels of the pro-inflammatory cytokine IL-6. Maciejczyk et al., demonstrated the application of salivary antioxidant potential assessed by FRAP-method as a marker of progression of chronic kidney disease in children [25]. Salivary biomarkers of oxidative stress were also investigated in children with chronic kidney disease [28]. 

Present data concern a pilot study with an accent on the potentials of saliva as a diagnostic tool. Further investigations with definite tendency of increase of the number of participants suffering from the common health disorder of pyelonephritis will contribute to proper evaluation of mRNA levels of IL-6, MMP-8 and GSS as salivary biomarkers. 

## 4. Materials and Methods

### 4.1. Ethics

An informed consent was given and a declaration of informed consent was signed by parents or legal guardians of children or another person accompanying each child participating in the research. The University Medical Ethical Institutional Board of the Medical University of Varna approved the use of the collected salivary samples from children with diagnosed pyelonephritis and healthy controls for the purposes of our study.

### 4.2. Participants

Inclusion criteria:

1. Participants between 0–18 years of age.
2. Children with established pyelonephritis.
3. A control group of children without any common health disorders.

Exclusion criteria:

1. Patients of pyelonephritis older than 18 of age.
2. Patients of clinical manifestation of periodontal diseases, respectively children with loss of bone tissue, loss of clinical attachment level or formation of periodontal pockets.
3. At the time of sample collection, patients do not suffer from other inflammatory diseases.
4. Do not suffer from autoimmune disorders or malignant diseases.
5. No anamnestic data of allergic reactions’ background
6. Do not use immunosuppressive drugs, no corticosteroids’ application, no antihistamins’ medication.

Therapy of all of these patients includes antiobiotics of the groups of penicillins, cephalosporines, macrolides, as well as non-steroid anti-inflammatory drugs.

Our study included 28 children suffering from pyelonephritis. A control group of nine children with no common health disorders was also included in the investigation. The patients were hospitalized at the Department of Pediatrics of St. Marina University Hospital. The gender distribution was as follows: 17 of the patients were female and 11 were male. A total number of four male and five female healthy children represented the control group. The healthy participants were outpatients of the University Dental Medicine Center at the Faculty of Dental Medicine, Medical University-Varna. Anamnestic data of each patient was obtained from their individual medical history.

### 4.3. Salivary Sample Collection

The procedure of saliva sample collection was performed at the bed of the patient individually. Healthy children were asked to provide saliva in outpatient conditions at the dental office. The samples of unstimulated whole saliva were collected in sterile DNase- and RNase-free collection tubes, frozen immediately on dry ice, and stored at −80 °C until further analysis. The saliva specimen collection was carried out in the time interval between 9.00 a.m. and 11.30 a.m. All the participants in the investigation were instructed to properly brush their teeth just before the sample collection. Frozen saliva was stored for a period of approximately 18 months.

### 4.4. Urine Sample Collection

For the planned urine assays are taken morning urine samples. There is a requirement to be provided second portion maximum clear urine samples. A small amount of urine had to be collected into individual sterile container. Immediately after urine collection, the container was tightly closed. 

### 4.5. Total RNA Extraction from Saliva and RT-qPCR Analysis 

Total RNA extraction was conducted using 200 µL unstimulated whole saliva and the TRIzol RNA extraction method (Ambion, Austin, TX, USA). Evaluation of RNA concentration and purity was performed spectrophotometrically using Synergy 2 Multi-Detection Microplate Reader (BioTek, Winooski, VT, USA). Before conducting RT-PCR DNase, treatment of total RNA was performed using DNase I recombinant kit (Roche Diagnostics GmbH, Mannheim, Germany) following the manufacturer’s instructions. For reverse transcription reaction 100 ng DNase-treated total RNA was added and cDNA was synthesized using RevertAid First Strand cDNA Synthesis Kit (ThermoScientific, Waltham, MA, USA) with oligo (dT)18 primer. Primers for real-time PCR were commercially synthesized (Sigma-Aldrich, Taufkirchen, Germany). Primer sequences were as follows: human actin beta forward 5′-CTGGAACGGTGAAGGTGACA-3′, reverse 5′-AAGGGACTTCCTGTAACAATGCA-3′; human IL-6 forward 5′-GGCACTGGCAGAAAACAACC-3′, reverse 5′-GCAAGTCTCCTCATTGAATCC-3′; human MMP-8 forward 5′-GCAACCCTATCCAACCTACTG-3′, reverse 5′-CATCCTCAGCTACAAAGAGTCG-3′; human GSS forward 5′-TACGGCTCACCCAATGCTC-3′, reverse 5′-GCACGCTGGTCAAATATGTTTC-3′. SYBR Green qPCR analysis was performed using AccuPower^®^ 2xGreenStarTM qPCR Master Mix (Bioneer, Oakland, CA, USA). Reactions were performed in 96 well plates under the following conditions: predenaturation and enzyme activation 95 °C/10 min, denaturation 95 °C/30 s, annealing 60 °C/30 s, extension 72 °C/30 s, 45 cycles. Each gene was analyzed in triplicate for all samples. Ct values were detected using ABI PRISM 7500 software (Applied Biosystems, Waltham, MA, USA). Gene expression levels were calculated using the 2^−∆∆Ct^ method [67] and expressed as relative units (RUs) as compared to the control group where the level of gene expression of interest was considered to be equal to 1. Data are presented as mean ± standard error of mean (SEM). Actin beta was used as endogenous control.

### 4.6. Para-Clinical Parameters of Proteins, Blood and Leucocytes Measurement in Urine Sample

For the hospitalized participants into the study has been implemented the routine chemical method of identification of proteins in urine samples. By means of a quantitative microscope, urine test were evaluated the levels of red blood cells and white blood cells. The microscope tests were performed after centrifugation of urine samples and the obtained sediment was used to examine the presence of white and/or red blood cells. 

### 4.7. Statistical Analysis

Statistical analysis t-test was performed using Microsoft Excel Office 2007 software, *p*-values < 0.05 were considered statistically significant. We used a correlation analysis with calculation of Pearson’s coefficient. The latter assesses the significance of linear interrelation between two variables. The values of Pearson’s coefficient in the range between 0.00 and 0.25 correspond to slight correlation. The values in the range from 0.26 to 0.49 are associated with moderate correlation. Levels of the coefficient from 0.50 to 0.69 are related to considerable correlation. The values from 0.70 to 0.89 characterize strong correlation. The values from 0.90 to 1.00 are characteristic of a very strong correlation. 

## 5. Conclusions

Salivary IL-6, MMP-8 and GSS mRNA levels in combination with urine test analysis could be a useful diagnostic tool for the very distributed disorder of pyelonephritis in childhood.

## Figures and Tables

**Table 1:** Descriptive analysis of anamnestic data and para-clinical urine indicators for all 28 children with pyelonephritis.

Patient №	Urine Analysis Indicators			Family Anamnesis for Kidney Diseases	Occurrence/Recurrence of Kidney Disorder
	Proteins	Blood	Leucocytes		
12	trace < 30 mg/dL	250 Ery/µL	+++ 500 Leuc/µL	None	Primary patient (Initial outset of the kidney disease)
28	+ 30–100 mg/dL	50 Ery/µL Hemolysis	+++ 500 Leuc/µL	None	Chronical kidney disease, in condition of exacerbation
7	0 mg/dL	10 Ery/µL Non-Hemolysis	+75 Leuc/µL	Father—with kidney aplasia (lack of one of the kidneys); brother- with significant bacteriuria;	Primary patient (Initial outset of the kidney disease)
17	trace < 30 mg/dL	50 Ery/µL Non-Hemolysis	25 Leuc/µL	None	Primary patient (Initial outset of the kidney disease)
4	0 mg/dL	negative	25 Leuc/µL	None	Primary patient (Initial outset of the kidney disease)
16	0 mg/dL	250 Ery/µL Non-Hemolysis	+ 75 Leuc/µL	None	Chronical kidney disease, in condition of exacerbation
11	trace < 30 mg/dL	50 Ery/µL Non-Hemolysis	+ 75 Leuc/µL	Sister—congenital anomaly of the excretory system (lack of one kidney)	Chronical kidney disease, in condition of exacerbation
23	0 mg/dL	10 Ery/µL Non-Hemolysis	25 Leuc/µL	None	Chronical kidney disease, in condition of exacerbation
19	+30–100 mg/dL	50 Ery/µL Hemolysis	+ 75 Leuc/µL	None	Primary patient (Initial outset of the kidney disease)
10	trace < 30 mg/dL	50 Ery/µL Non-Hemolysis	+ 75 Leuc/µL	None	Primary patient (Initial outset of the kidney disease)
6	trace < 30 mg/dL	10 Ery/µL Non-Hemolysis	25 Leuc/µL	None	Chronical kidney disease, in condition of exacerbation
5	trace < 30 mg/dL	250 Ery/µL	+ 75 Leuc/µL	None	Primary patient (Initial outset of the kidney disease)
2	trace < 30 mg/dL	50 Ery/µL Non-Hemolysis	+ 75 Leuc/µL	None	Primary patient (Initial outset of the kidney disease)
1	trace < 30 mg/dL	250 Ery/µL	+ 75 Leuc/µL	Mother with nephrolithiasis	Primary patient (Initial outset of the kidney disease)
24	trace < 30 mg/dL	50 Ery/µL Non-Hemolysis	+ 75 Leuc/µL	Father with non-diagnosed kidney disorder	Chronical kidney disease, in condition of exacerbation
27	+30–100 mg/dL	5-10 Ery/µL Hemolysis	+ 75 Leuc/µL	None	Primary patient (Initial outset of the kidney disease)
21	trace < 30 mg/dL	50 Ery/µL Non-Hemolysis	+ 75 Leuc/µL	None	Primary patient (Initial outset of the kidney disease)
25	trace < 30 mg/dL	50 Ery/µL Non-Hemolysis	+ 75 Leuc/µL	None	Primary patient (Initial outset of the kidney disease)
26	trace < 30 mg/dL	50 Ery/µL Hemolysis	+ 75 Leuc/µL	Aunt with kidney agenesia; cousin suffering from pyelonephrithis	Chronical kidney disease, in condition of exacerbation
3	trace < 30 mg/dL	10 Ery/µL Non-Hemolysis	25 Leuc/µL	None	Chronical kidney disease, in condition of exacerbation
8	trace < 30 mg/dL	50 Ery/µL Non-Hemolysis	25 Leuc/µL	Grandfather and father suffering from nephrolithiasis	Chronical kidney disease, in condition of exacerbation
9	trace < 30 mg/dL	25 Ery/µL Hemolysis	25 Leuc/µL	Grand-grandfather and grandfather suffering from nephrolithiasis	Chronical kidney disease, in condition of exacerbation
14	+30–100 mg/dL	50 Ery/µL Hemolysis	+ 75 Leuc/µL	Aunt suffering from renal failure; mother with diagnosed pyelonephritis	Chronical kidney disease, in condition of exacerbation
22	trace < 30 mg/dL	250 Ery/µL	+++ 500 Leuc/µL	None	Chronical kidney disease, in condition of exacerbation
20	trace < 30 mg/dL	5–10 Ery/µL Hemolysis	25 Leuc/µL	None	Primary patient (Initial outset of the kidney disease)
13	+30–100 mg/dL	5–10 Ery/µL Hemolysis	+ 75 Leuc/µL	None	Primary patient (Initial outset of the kidney disease)
15	trace < 30 mg/dL	50 Ery/µL Non-Hemolysis	25 Leuc/µL	None	Chronical kidney disease, in condition of exacerbation
18	trace < 30 mg/dL	250 Ery/µL	+ 75 Leuc/µL	Grandmother suffering from nephrolithiasis	Chronical kidney disease, in condition of exacerbation

Each patient identified as suffering from chronical pyelonephritis is in condition of exacerbation at the moment of registration of the determined indicators. Patients included in Table 1 are presented in a descending order based on the values of the indicator salivary IL-6. IL-6 is considered as an essential marker of acute inflammatory processes, as well as of exacerbation of chronical inflammation.

**Table 2:** Coefficient of Correlation by Pearson’s between Salivary Biomarkers and Para-clinical Indicators from Urine Assay.

	Leu	*p*	Blood	*p*	IL-6	*p*	GSS	*p*	MMP-8	*p*
**Leu**	-	-	0.45	<0.05	0.43	<0.05	0.54	<0.01	0.72	<0.001
Blood	0.45	<0.05	-	-	0.15	ns	0.20	ns	0.30	ns
IL-6	0.43	<0.05	0.15	ns	-	-	0.75	<0.001	0.75	<0.001
GSS	0.54	<0.01	0.20	ns	0.75	<0.001	-	-	0.86	<0.001
MMP-8	0.72	<0.001	0.30	ns	0.75	<0.001	0.86	<0.001	-	-

The values of Pearson’s coefficient in the range between 0.00 and 0.25 correspond to slight correlation. The values in the range from 0.26 to 0.49 are associated with moderate correlation. Levels of the coefficient from 0.50 to 0.69 are related to considerable correlation. The values from 0.70 to 0.89 characterize strong correlation. The values from 0.90 to 1.00 are characteristic of a very strong correlation. *p*-values < 0.05 were considered statistically significant.
